# The Effect of *Lactiplantibacillus plantarum* I-Enriched Diet on the Phenolic Content and Antioxidant Capacity of Queen Scallop (*Aequipecten opercularis* Linnaeus, 1758) Extracts

**DOI:** 10.3390/microorganisms11112723

**Published:** 2023-11-07

**Authors:** Ines Kovačić, Petra Burić, Ante Žunec, Josipa Bilić, Anamarija Prgić, Iva Čanak, Neven Iveša, Mauro Štifanić, Jadranka Frece

**Affiliations:** 1 Faculty of Educational Sciences, Juraj Dobrila University of Pula, Zagrebačka 30, 52100 Pula, Croatia; ikovacic@unipu.hr; 2 Faculty of Natural Sciences, Juraj Dobrila University of Pula, Zagrebačka 30, 52100 Pula, Croatia; azunec@unipu.hr (A.Ž.); neven.ivesa@unipu.hr (N.I.); mauro.stifanic@unipu.hr (M.Š.); 3 METRIS Research Centre, Istrian University of Applied Sciences, Preradovićeva 9D, 52100 Pula, Croatia; jbilic@iv.hr; 4 Faculty of Science, University of Zagreb, Rooseveltov trg 6, 10000 Zagreb, Croatia; anamarijaprgic2@gmail.com; 5 Faculty of Food Technology and Biotechnology, University of Zagreb, Pierottijeva 6, 10000 Zagreb, Croatia; iva.canak@pbf.unizg.hr (I.Č.); jadranka.frece@pbf.unizg.hr (J.F.)

**Keywords:** probiotic, *Lactiplantibacillus plantarum* I, antioxidant capacity, phenolic content

## Abstract

The use of probiotics in the diet of bivalves poses a great potential in aquaculture as an alternative to antibiotics. The aim of this study was to assess the effect of *Lactiplantibacillus plantarum* I on the phenolic content and antioxidant capacity (AC) of queen scallop extracts after one month of feeding. Total phenols (TP) ranged from 28.17 ± 3.11 to 58.58 ± 8.57 mg GAE/100 g, total non-flavonoids (TNF) from 23.33 ± 3.66 to 36.56 ± 9.91 mg GAE/100 g, and total flavonoids (TF) from 10.56 ± 5.57 to 30.16 ± 1.69 mg CE/100 g. AC was assessed via three different methods: the ferric-reducing ability of plasma assay (FRAP), 2,2′-azino-bis (3-ethylbenzothiazoline-6-sulfonic) acid assay (ABTS), and 2,2-diphenyl-1-picryhydrazyl assay (DPPH). FRAP values ranged from 0.13 ± 0.03 to 0.17 ± 0.02 µM AA/g, ABTS from 0.68 ± 0.11 to 2.79 ± 0.34 µM AA/g, and DPPH from 1.75 ± 0.17 to 2.98 ± 0.53 µM AA/g. Among all extracts, the best phenolic content and AC were observed in water extracts from queen scallops. The bivalves treated with the *Lactiplantibacillus plantarum* I-enriched diet showed higher AC according to the FRAP assay in all extracts. A significant correlation was observed between AC and TP and TNF in control and *Lactiplantibacillus plantarum* I-treated scallops.

## 1. Introduction

Probiotics are a food supplement with live commensal microorganisms that can modulate the ecosystem and population of the gut microbiota and boost host immunity when administered in a proper quantity [1]. Lactic acid bacteria (LAB) are considered one of the essential health-promoting bacteria. They are used for the treatment of diverse types of diseases or disorders in humans, as monotherapies or combined [2,3,4]. LAB produce a broad range of defense mechanisms with extensive potency of establishment and production of helpful agents. Additionally, they are clinically used as biocontrol agents [2].

Over the past two decades, extensive research has been undertaken to assess the probiotic attributes of various genera and species [5,6]. These studies collectively underscore the pivotal role of LABs, in conjunction with other bacterial constituents of the indigenous microbiota, in bolstering the health and overall well-being of aquatic organisms, encompassing both fish and shellfish. Among several probiotic bacterial species, a body of literature details the advantageous influence of *Lactiplantibacillus plantarum* (formerly known as *Lactobacillus plantarum*) on marine organisms [7,8,9]. Scientists are trying to isolate probiotic cultures from marine organisms, culture them in vitro, and further supply them as food supplements in the aquaculture of commercially important species [9,10,11,12,13,14,15,16]. In recent years, the use of probiotics in the aquaculture industry has seen significant growth due to their distinctive advantages and versatile applications for both fish and shellfish [17,18,19]. Aquaculture has embraced probiotics for enhancing fish and shellfish growth, bolstering disease resistance, boosting immunity, and maintaining water quality [20]. Several studies have investigated feeding of queen scallop (*Aequipecten opercularis)*, lion’s paw scallop (*Nodipecten subnodosus*), oyster (*Pteria sterna*), Pacific oyster (*Crassostrea gigas*), juvenile turbot (*Scophthalmus maximus*), Nile tilapia (*Oreochromis niloticus*), and common carp (*Cyprinus carpio*) supplemented with *Lactiplantibacillus plantarum*, and the results have shown improved digestion and nutrient absorption, leading to enhanced growth rates, increased disease resistance, and overall vitality in shellfish and fish populations [9,11,13,20]. Moreover, probiotics serve as cost-effective and environmentally safe alternatives to antibiotics, making them suitable substitutes in aquafeed [21]. These probiotics are added to diets to enhance productivity by improving the health and antioxidative capacity of marine species [22,23,24,25].

In the past decade, there have been numerous reports on the antioxidants and total antioxidant capacity found in marine organisms [26,27,28,29,30,31,32,33,34,35,36,37,38,39,40,41,42]. Wide varieties of bivalve molluscs (*Amusium japonicum taiwanicum*, *Anadara granosa*, *Atrina pectinata*, *Meretrix meretrix*, *Perna viridis*, *Ruditapes philippinarum*, and *Scapharca kagoshimensis*) have been found to have a high antioxidant potential [28,29,43]. Antioxidants can be enzymatic or non-enzymatic in nature. Non-enzymatic antioxidants include vitamins (A, C, E), selenium, polyunsaturated fatty acids, total phenols (TP), total flavonoids (TF), and total non-flavonoids (TNF), which were also found in marine organisms [6,30,31]. To evaluate the antioxidant status of an organism, the measurement of antioxidant capacity (AC) is often used [44,45,46,47,48]. The AC value integrates the response of the tested organism to reduce the free radicals or reactive oxygen species (ROS) via the antioxidant system [48]. ROS are molecules consisting of atoms missing one or more electrons, thus being highly unstable and reactive [29]. In order to achieve stability, these molecules attract the missing electrons, whereby they often attack the main cellular components, such as lipids, proteins, and nucleic acids [32]. Damage to these crucial cell components can cause numerous irreversible changes, such as mutations that may lead to various diseases (degenerative diseases, diabetes, atherosclerosis, Parkinson’s disease, inflammatory bowel disease, etc.) [1,2] and, finally, to the death of the organism [49,50,51,52,53].

The use of marine bivalves in promoting human health is being explored in different shellfish species. Bivalve molluscs represent a natural source of animal protein and natural omega-3 polyunsaturated fatty acids in the everyday human diet [54,55,56]. In 2018, the Food and Agriculture Organization (FAO) [57] reported that in 1.5 billion people around the world, roughly 15% of the animal protein intake comes from bivalve farming. This is the reason for the rapid growth of shell aquaculture in recent decades.

The queen scallop (*Aequipecten opercularis* Linnaeus, 1758) is an edible marine bivalve from the Pectinidae family that is of great commercial importance for the eastern Atlantic, Mediterranean, and Adriatic Seas [58,59,60]. For more than 100 years, Pectinids have been commercially exploited in Europe [60,61]. The European catch of the queen scallop ranged from 10,000 tons in the mid-1970s, reaching 60,000 tons in 2012, with the Irish Sea and the Isle of Man contributing to ~80% of total landings [61]. Queen scallops reach their commercially profitable size in their second year, when they become vulnerable to fishing and environmental change [60,61]. Due to the high market demand for queen scallop, overfishing, and high market prices of species, such as the related *Pecten jacobaeus* (L.) in the Mediterranean, queen scallop is of great interest for the higher marketable demand and as an alternative shellfish species for cultivation in the Adriatic Sea [61].

The aim of this study was to determine the phenolic content and AC in the tissue homogenate of the adult scallop *A. opercularis* sampled in the Medulin Bay area. Spectrophotometric methods were used to compare the phenolic content (TP, TF, TNF) and AC in water, 90% methanol, and 70% ethanol homogenates of the total bivalve tissue. The results obtained from individuals treated with probiotic culture *Lactiplantibacillus plantarum* I were compared to the untreated bivalves. This research will contribute to a better understanding of the AC of these commercially important bivalves. Furthermore, it will provide consumers with more data regarding marine bivalves that might be of great commercial value for human health.

## 2. Materials and Methods

### 2.1. Microorganisms

Strain *Lactiplantibacillus plantarum* I used in this study was isolated from the digestive system (intestine) of live queen scallops (*A. opercularis*), identified, and characterized in our previous research [9]. The strain is permanently deposited in the collection of microorganisms of the Laboratory for General Microbiology and Food Microbiology at the Department of Biochemical Engineering, Faculty of Food Technology and Biotechnology, University of Zagreb, Croatia.

### 2.2. Reagents

All reagents and solvents used for TP, TNF, TF, and AC analyses were of adequate analytical grade and were obtained from Kemika (Zagreb, Croatia), Merck KGaA and AppliChem GmbH (Darmstadt, Germany), Acros Organics (Geel, Belgium), Sigma Aldrich GmbH (Taufkirchen, Germany), and Fisher Scientific (Loughborough, UK). Reference substances were obtained from Sigma Aldrich (St. Louis, MO, USA) and were HPLC-graded. Ferric-2,4,6-tripyridyl-D-triazine (FRAP), 2,2-diphenyl-1-picryhydrazyl (DPPH), and 2,2′-azino-bis-(3-ethylbenzothiazoline-6-sulfonic) acid (ABTS) complexes were used for spectrophotometric analysis of the antioxidant activity.

### 2.3. Sampling and Keeping

The queen scallop *A. opercularis* (Linnaeus, 1758) was collected in January 2021, from a place located two nautical miles southeast of the Albanež shoal (Municipality of Medulin, Croatia), within the E2 fishing zone (44°43′58.49″ N, 13°56′48.94″ E). Sampling was performed by local fishermen using a fishing vessel bottom-trawling net from a depth of ~50 m. The individual queen scallops (n = 120) used in this study were 50.5 ± 4.0 mm in shell length and 49.6 ± 3.1 mm in shell width. After sampling, the bivalves were transferred to the Aquarium Pula facility, where they were placed in a circular basin (V = 1900 L) with a flow-through seawater system (200 L/h). During the acclimatization period, as well as during the experiment, the bivalves were fed daily, as previously described by Čanak et al. [9]. Briefly, the scallops were fed daily with a mix of algae cultures consisting of *Tetraselmis* sp. (Chlorophyta; 5 × 10^5^ cells/mL), *Nannochloropsis* sp. (Eustigmatophyceae; 30 × 10^5^ cells/mL), and *Phaeodactylum* sp. (Bacillariophyta; 12 × 10^5^ cells/mL).

### 2.4. Preparation of Wet Biomass for Supplementation and Feeding

Strain *Lactiplantibacillus plantarum* I was cultured in MRS broth (Biolife, Milan, Italy) at 37 °C overnight. Bacterial cells were collected using aseptic centrifugation at 6440 rcf for 10 min and then washed twice with physiological saline. The cells were finally resuspended in sterile physiological saline. The total viable count (TVC) of *Lactiplantibacillus plantarum* I was determined using the standard dilution method on MRS agar (Biolife, Milan, Italy), followed by incubation at 37 °C for 24–48 h. The final count yielded 10^9^ viable bacterial cells of *Lactiplantibacillus plantarum* I per gram of wet biomass.

### 2.5. Experimental Design

After a week of acclimatization, 60 individuals were placed in two separate basins with 190 L of seawater per basin (30 individuals per basin). The control basin was fed only with phytoplankton, while in the experimental basin, probiotic culture was added to the same final density. The second experiment (control + LAB-fed scallops) was estimated as replicants at the same time in the Aquarium Pula. The cultures were kept for 30 days in the same conditions of temperature, pH, and dissolved oxygen (measured with a Hanna HI98193 multiparameter probe). During the morning feeding period, which lasted for six hours, the water flow in the aquarium was deliberately kept closed. To eliminate any accumulated excess food and feces, the bottom of the aquarium was carefully siphoned. After a month, the scallop tissue was collected as composite samples of ten bivalves and immediately stored at −20 °C for further analysis. Each sample was prepared in two subsamples and all measurements were performed in triplicate.

### 2.6. Determination of Total Phenols, Flavonoids, Non-Flavonoids, and Total Antioxidant Capacity

The ratio of scallop tissue and the selected solvent (water, 90% methanol, or 70% ethanol) was fixed at 1:3. Roughly 0.5 g of the bivalve tissue samples was mixed with 1.5 mL of distilled water, 90% methanol, or 70% ethanol. The solvents for this work were chosen based on their polarities and they ensured different extractions of the compounds, based on which the AC was compared [58]. The mixture was then homogenized using an OV5 Homogenizer (VELP Scientifica Srl, Usmate Velate, Italy) for 5 min. The homogenate was then centrifuged (10,000 rpm, 30 min at 4 °C) with a 5430 R-High-Speed Centrifuge (Eppendorf SE, Hamburg, Germany).

TP was determined spectrophotometrically, as previously described by Singleton et al. [62]. Briefly, by adding the Folin–Ciocalteu reagent to the reaction mixture, it reacted with the phenoxide ion from the sample, forming a blue color solution. The absorbance was read at 765 nm two hours after adding the reagent to the reaction. TF were determined according to the method of Martins et al. [45]. Briefly, by adding aluminum chloride to the reaction mixture, a red color was formed, which was read at 510 nm. For determining the content of TNF, the method of Ough and Amerine [63] was used. Briefly, flavonoids were first removed from the samples using formaldehyde. Formaldehyde reacts with flavonoids in an acidic medium and causes their sedimentation, after which the same method described above for TP was applied. The results were expressed in mg gallic acid equivalent per 100 g of shell tissue (mg GAE/100 g of tissue) and in mg of catechin equivalents per 100 g of shell tissue (mg CE/100 g of tissue). The results of TP and TNF were calculated according to the calibration curve for gallic acid in water (y = 0.001x, y = absorbance at 765 nm, x = concentration of gallic acid mg/L, R^2^ = 0.9995), 70% ethanol (y = 0.0004x, y = absorbance at 765 nm, x = concentration of gallic acid mg/L, R^2^ = 0.9915), and 90% methanol (y = 0.0009x, y = absorbance at 765 nm, x = concentration of gallic acid mg/L, R^2^ = 0.9952). The results of TF were calculated according to the calibration curve for catechin in water (y = 0.0036x, y = absorbance at 510 nm, x = concentration of catechin mg/L, R^2^ = 0.9999), 70% ethanol (y = 0.0024x, y = absorbance at 510 nm, x = concentration of catechin mg/L, R^2^ = 0.9933), and 90% methanol (y = 0.0025x, y = absorbance at 510 nm, x = concentration of catechin mg/L, R^2^ = 0.9991).

The AC of bivalve tissue was determined as previously described by Poljuha et al. [60] using three methods. The results were expressed in µM ascorbic acid equivalent per g of shell tissue (µM AA/g of tissue). The ABTS method was carried out on the principle that the blue–green ABTS radical cation and potassium persulfate are added to the solutions in order to cause oxidation. The reduction of the ABTS was measured at 734 nm as the disappearance of the color. The results of antioxidant capacity measured by the ABTS assay were calculated according to the calibration curve for ascorbic acid in water (y = 52.665x, y = percentage of inhibition of ABTS radical, x = concentration of ascorbic acid mmol/L, R^2^ = 0.9968), 70% ethanol (y = 42.657x, y = percentage of inhibition of ABTS radical, x = concentration of ascorbic acid mmol/L, R^2^ = 0.9958), and 90% methanol (y = 43.11x, y = percentage of inhibition of ABTS radical, x = concentration of ascorbic acid mmol/L, R^2^ = 0.9969). The experiments were carried out in triplicate. The FRAP method is based on the reduction of iron as a part of the Fe (TPTZ)^3+^ complex, into the ferrous form of Fe (TPTZ)^2+^ in the presence of antioxidants, resulting in an intense blue color for which absorbance was measured at 593 nm. The reaction took place in an acidic medium (pH = 3.6) in order to preserve the solubility of iron and increase the redox potential. The results of antioxidant capacity measured with FRAP were calculated according to the calibration curve for ascorbic acid in water (y = 1.6851x, y = absorbance at 593 nm, x = concentration of ascorbic acid mmol/L, R^2^ = 0.9822), 70% ethanol (y = 1.8485x, y = absorbance at 593 nm, x = concentration of ascorbic acid mmol/L, R^2^ = 0.9543), and 90% methanol (y = 1.6927x, y = absorbance at 593 nm, x = concentration of ascorbic acid mmol/L, R^2^ = 0.9822). The experiments were carried out in triplicate. The DPPH method is based on the reduction of the DPPH radical in the presence of antioxidants, during which the purple color changes to yellow. The absorbance color was measured at 517 nm after 60 min. The results of antioxidant capacity measured by the DPPH assay were calculated according to the calibration curve for ascorbic acid in water (y = 59.688x, y = percentage of inhibition of DPPH radical, x = concentration of ascorbic acid mmol/L, R^2^ = 0.9812), 70% ethanol (y = 48.506x, y = percentage of inhibition of DPPH radical, x = concentration of ascorbic acid mmol/L, R^2^ = 0.9943), and 90% methanol (y = 60.757x, y = percentage of inhibition of DPPH radical, x = concentration of ascorbic acid mmol/L, R^2^ = 0.997). The experiments were carried out in triplicate.

All measurements were performed using a NanoPhotometer P300 spectrophotometer (Implen GmbH, München, Germany).

### 2.7. Statistical Analysis

The Statistica 9.0 software (StatSoft Inc., Tulsa, OK, USA) was used for statistical analysis. The difference in the amount of TP, TF, TNF, and AC in water, 90% methanol, and 70% ethanol tissue homogenates of control and LAB-fed scallops were determined using the *t*-test. To investigate the influence of diet and solvents on TP, TF, TNF, and AC, analysis of variance (factorial ANOVA) was used. The data are shown as mean ± SD and the significant difference among the solutions used. Pearson’s coefficient at the 0.05 level was used to determine the relation between the contents of bioactive compounds and antioxidative capacity.

## 3. Results

### 3.1. Phenolic Content in Queen Scallop Extracts

**Figure 1:**
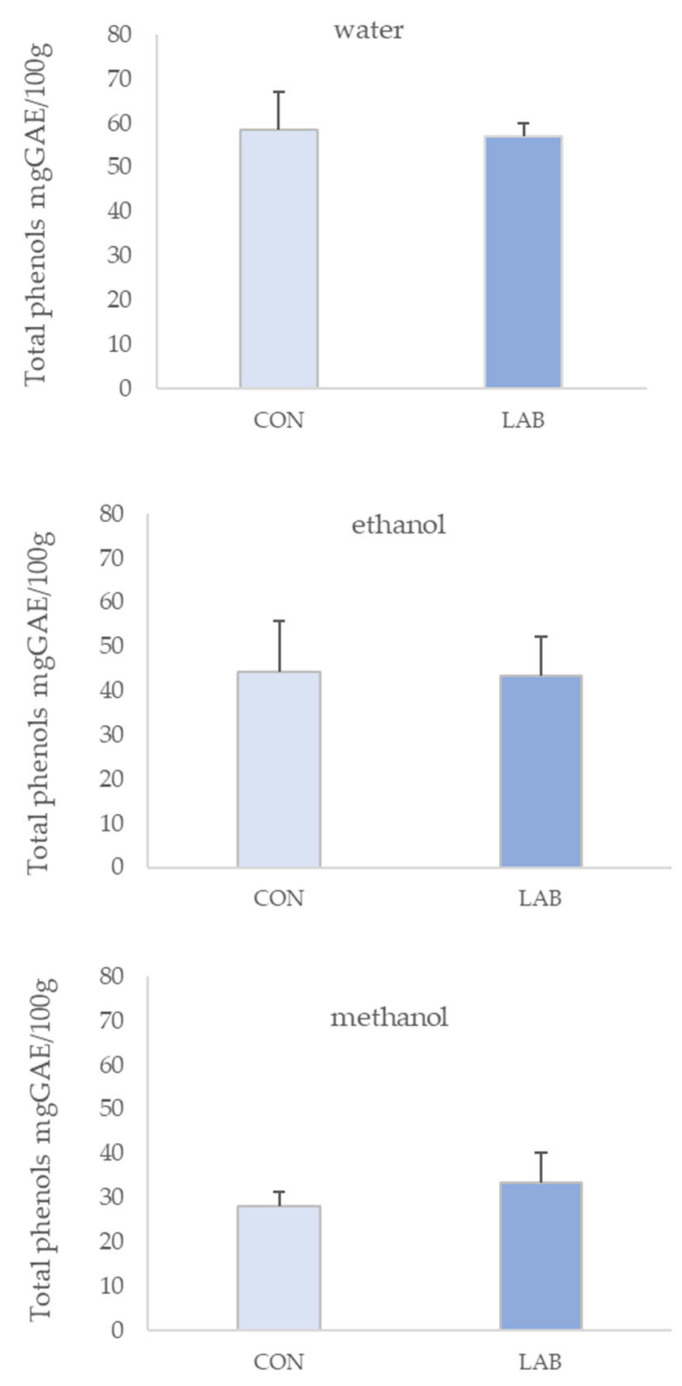
Total phenols (mean ± SD) in water, 70% ethanol, and 90% methanol extracts of control scallops (CON) and scallops fed with a *Lactiplantibacillus plantarum* I (LAB)-enriched diet.

TP obtained in different solvent extracts of queen scallop is shown in Figure 1. TP ranged from 28.17 ± 3.11 to 58.58 ± 8.57 mg GAE/100 g of queen scallop extracts from the control group (fed only with phytoplankton) and from 33.25 ± 6.78 to 56.93 ± 3.07 mg GAE/100 g in queen scallop extracts whose diet was enriched with LAB, depending on the solvent type. The highest TP content was determined in the control (58.58 ± 8.57 mg GAE/100 g) and LAB-enriched (56.93 ± 3.07 mg GAE/100 g) queen scallop extracts where water was used as the solvent.

**Figure 2:**
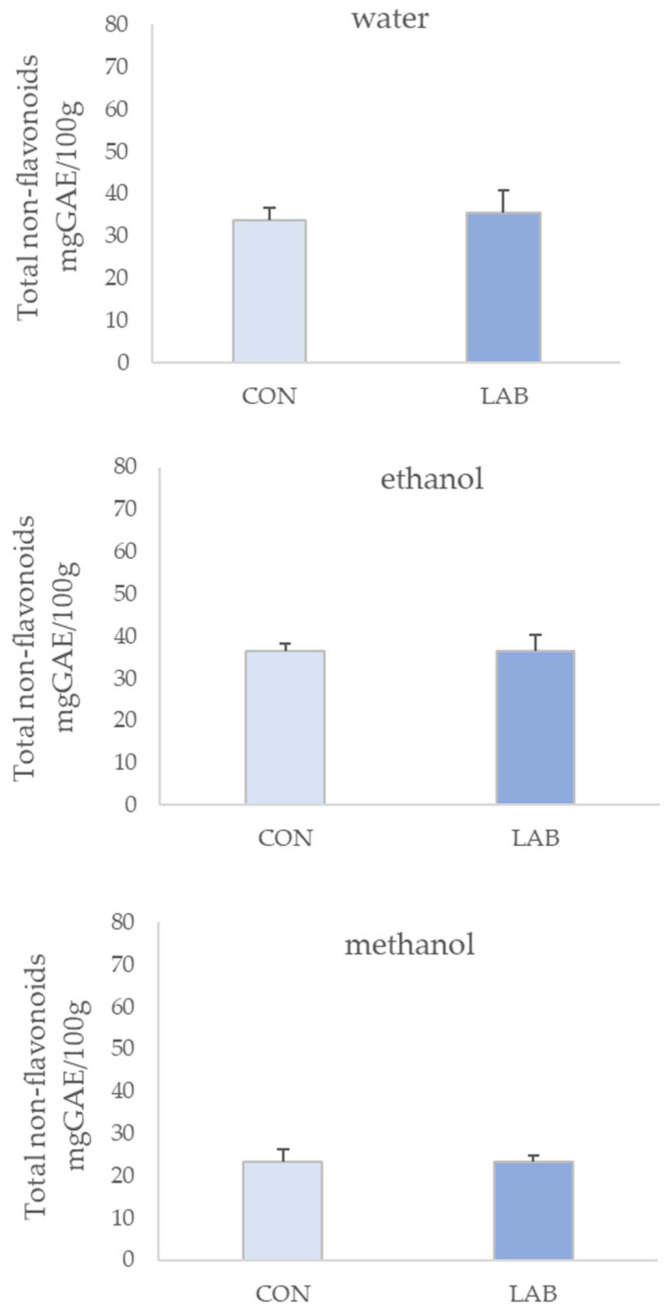
Total non-flavonoids (mean ± SD) in water, 70% ethanol, and 90% methanol extracts of control scallops (CON) and scallops fed with a *Lactiplantibacillus plantarum* I (LAB)-enriched diet.

The amount of TNF determined in different solvents of queen scallop extracts is shown in Figure 2. TNF ranged from 23.33 ± 3.66 to 36.56 ± 9.91 mg GAE/100 g in queen scallop extracts from the control basin and from 23.33 ± 1.68 to 35.55 ± 5.23 mg GAE/100 g in queen scallop extracts where LAB was added to enrich the diet, depending on the solvent type. The highest TNF content was determined in 70% ethanol extracts of LAB-enriched (36.56 ± 9.91 mg GAE/100 g) and control (36.56 ± 6.71 mg GAE/100 g) queen scallops.

**Figure 3:**
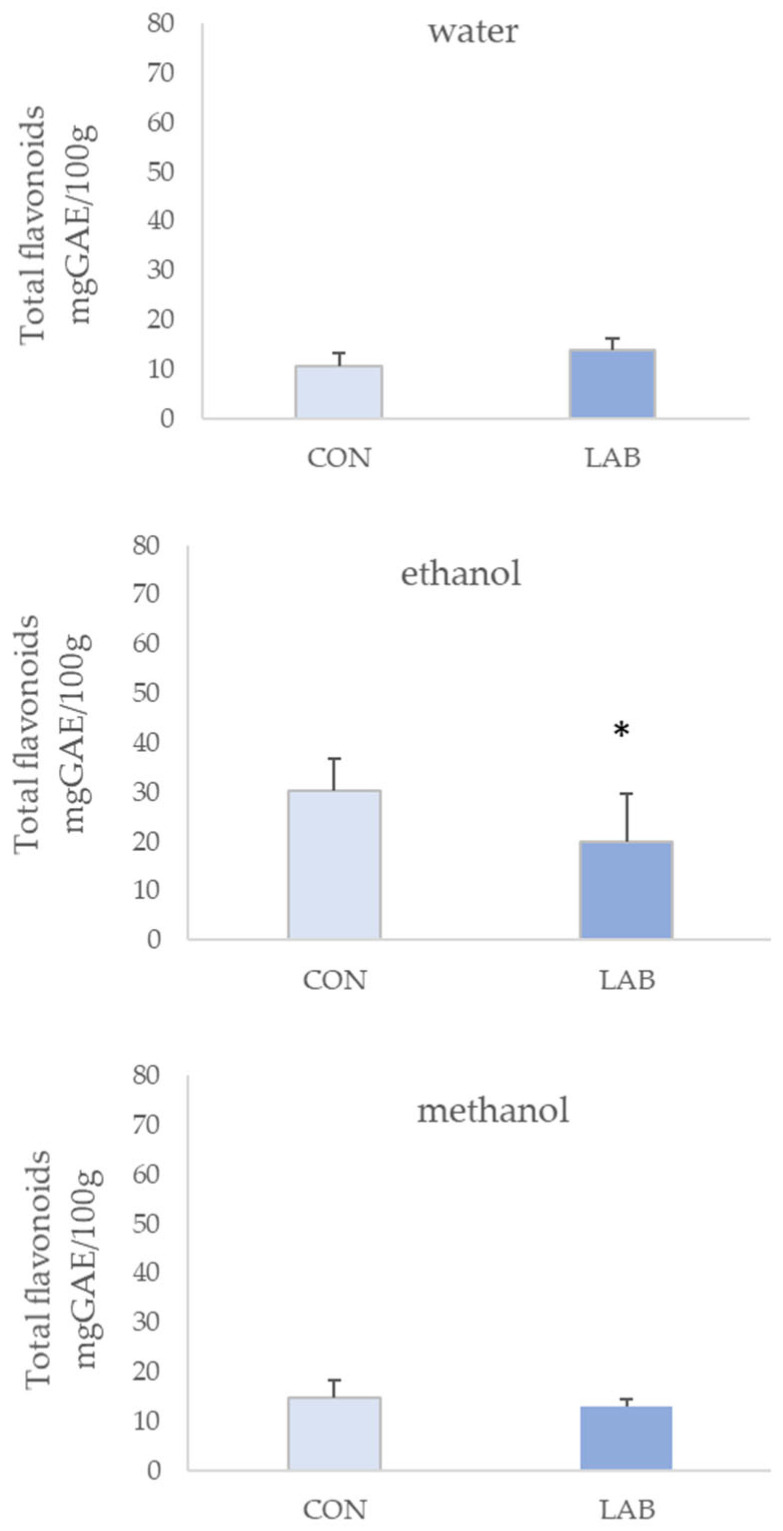
Total flavonoids (mean ± SD) in water, 70% ethanol, and 90% methanol extracts of control scallops (CON) and scallops fed with a *Lactiplantibacillus plantarum* I (LAB)-enriched diet. The asterisk indicates the statistical difference (* *p* < 0.05, *t*-test).

The level of TF measured in different solvent extracts of queen scallops is shown in Figure 3. TF ranged from 10.56 ± 2.58 to 30.17 ± 1.70 mg CE/100 g in queen scallop extracts from the control group and from 12.93 ± 1.45 to 19.84 ± 3.84 mg CE/100 g in queen scallop extracts whose diet was enriched with LAB, depending on the solvent type. The highest TF content was determined in 70% ethanol extracts from the control (30.17 ± 1.70 mg CE/100 g) and LAB-enriched (19.84 ± 3.83 mg CE/100 g) queen scallops.

The obtained results clearly indicate the noteworthy influence of the solvent used in the queen scallop extracts in terms of total phenolic concentrations. Results presented in Figure 1 show that water was the best solvent for extracting TP, while Figure 2 and Figure 3 present that 70% ethanol was the best solvent for extracting TNF and TF.

To emphasize the above results, the influence of the queen scallop diet and solvent used for the extraction of biologically active compounds on the concentrations of TP, TNF, and TF is presented in Table 1.

### 3.2. Antioxidant Capacity in Queen Scallop Extracts

**Figure 4:**
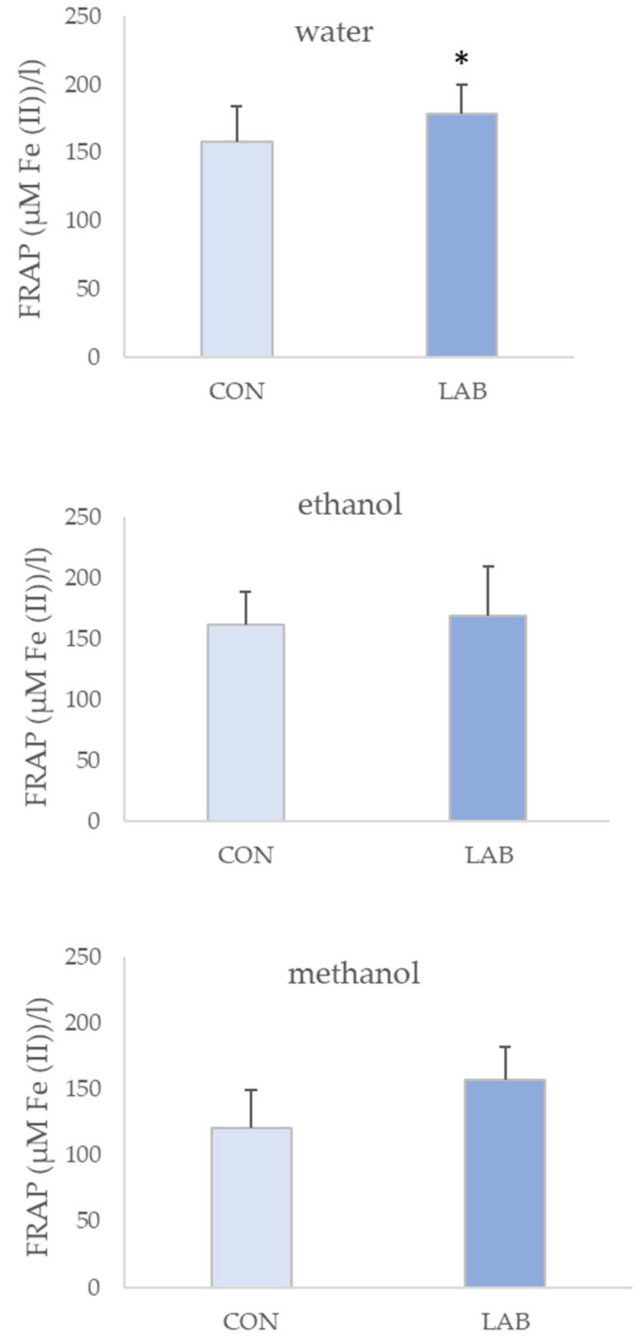
Antioxidant capacity (mean ± SD) in water, 70% ethanol, and 90% methanol extracts of control scallops (CON) and scallops fed with a *Lactiplantibacillus plantarum* I (LAB)-enriched diet, measured by the FRAP assay. The asterisk indicates the statistical difference (* *p* < 0.05, *t*-test).

All queen scallop extracts showed antioxidant potential. FRAP assay values in control queen scallops varied from 0.13 ± 0.03 µM AA/g in the 90% methanol extract to 0.16 ± 0.03 µM AA/g in water extracts (Figure 4). Queen scallops fed with the LAB-enriched diet had the highest FRAP values, with statistical significance only in 70% methanol extracts. Water extracts of queen scallops fed with the LAB-enriched diet had the highest FRAP values (0.18 ± 0.02 µM AA/g).

**Figure 5:**
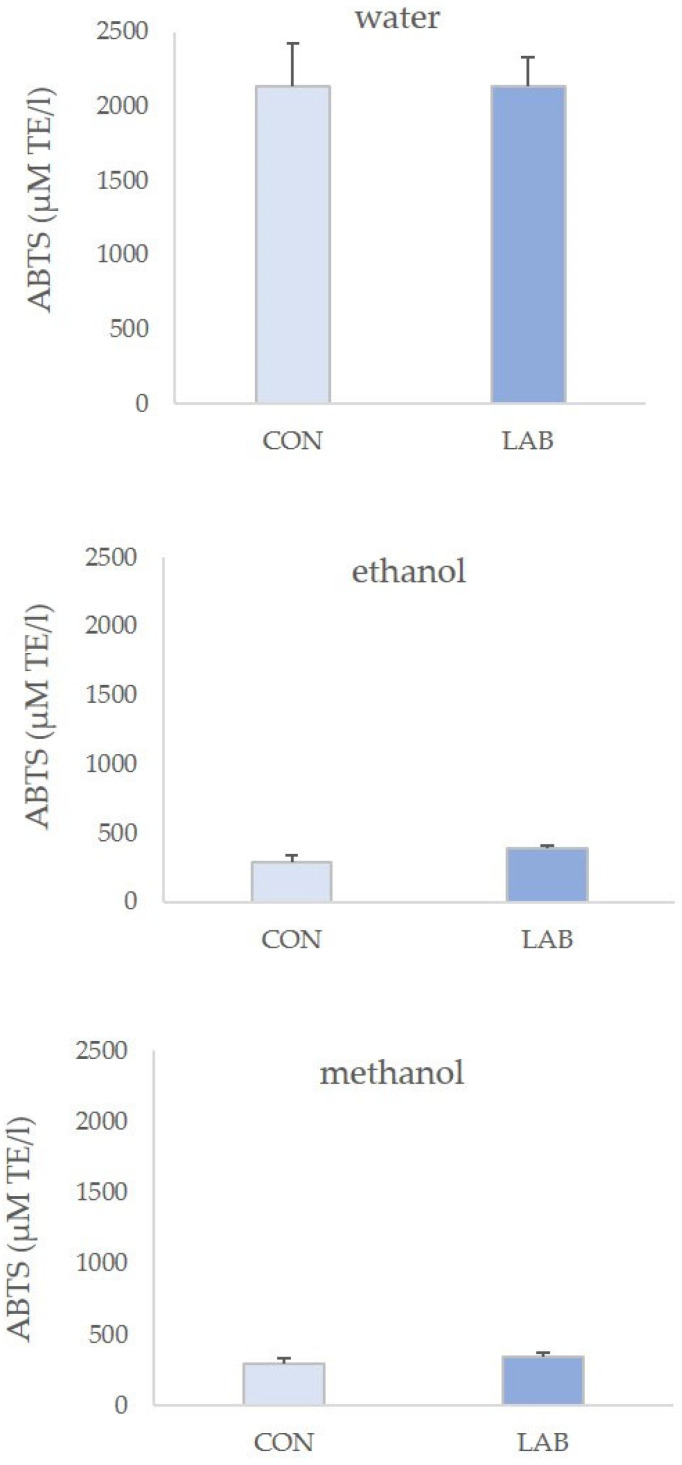
Antioxidant capacity (mean ± SD) in water, 70% ethanol, and 90% methanol extracts of control scallops (CON) and scallops fed with a *Lactiplantibacillus plantarum* I (LAB)-enriched diet, measured by the ABTS assay.

The highest antioxidative potential measured with the ABTS assay was displayed for the extracts that were prepared in water as the solvent (Figure 5). ABTS values in control queen scallops varied greatly, from 0.68 ± 0.12 µM AA/g in 70% ethanol extract to 2.80 ± 0.35 µM AA/g in water extract. Water extracts of queen scallops fed with the LAB-enriched diet had the lowest ABTS values in 70% ethanol extract (0.90 ± 0.06 µM AA/g) and the highest ABTS values in water extract (2.56 ± 0.24 µM AA/g).

**Figure 6:**
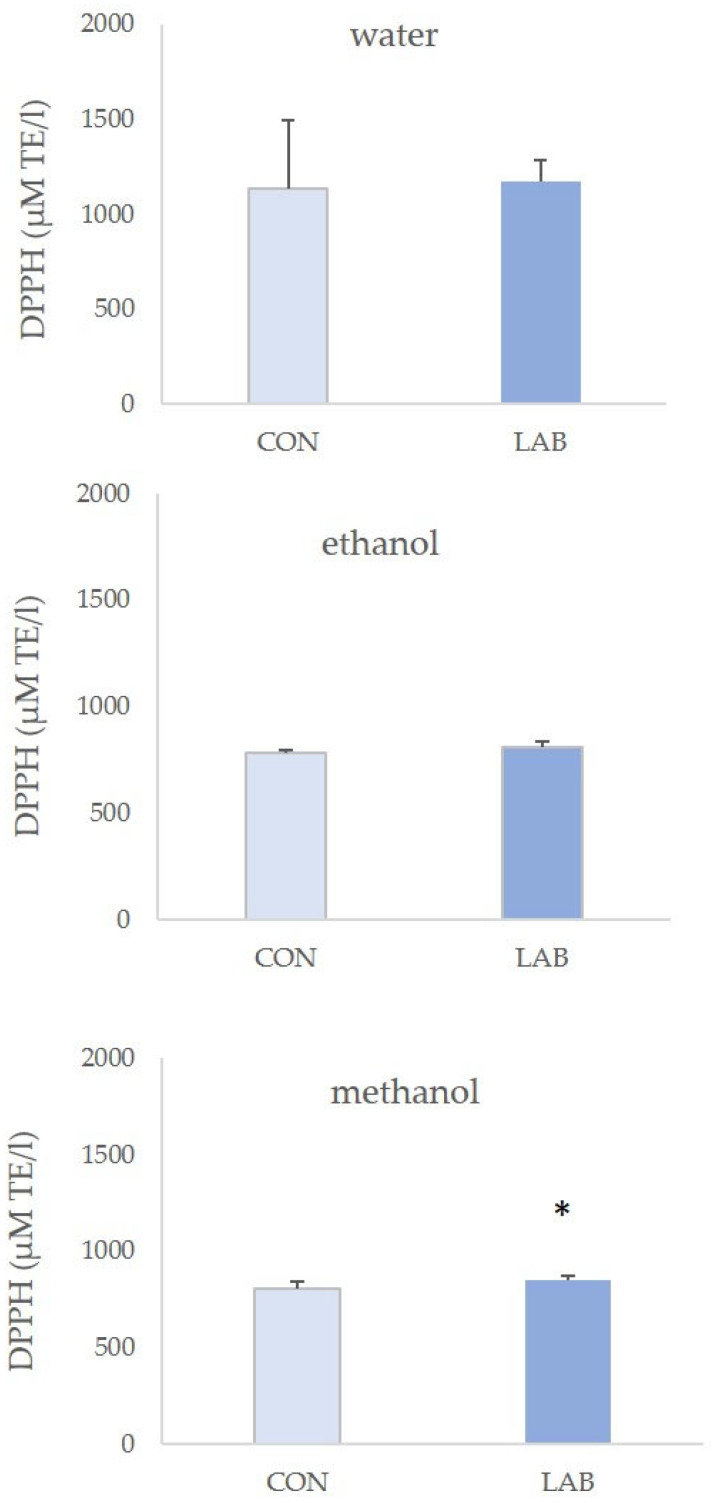
Antioxidant capacity (mean ± SD) in water, 70% ethanol, and 90% methanol extracts of control scallops and scallops fed with a *Lactiplantibacillus plantarum* I-enriched diet, measured by the DPPH assay. The asterisk indicates the statistical difference (* *p* < 0.05, *t*-test).

If all the antioxidant methods are compared, the DPPH method had the highest results (Figure 6). The highest antioxidative potential measured in this assay was from water extracts of control queen scallops (2.98 ± 0.53 µM AA/g) when compared to other solvents. DPPH assay values in LAB-fed queen scallops varied from 2.26 ± 0.08 µM AA/g in 70% ethanol extract to 1.75 ± 0.17 µM AA/g in water extract.

According to the results presented in Table 2, the diet and solvents used in the experiment had a direct significant effect on the antioxidant capacity measured with all three methods.

Correlation analysis was used to determine the relationships between the phenolic content and antioxidant capacities. Table 3 shows the correlation coefficients between TP, TNF, TF, and antioxidant capacity measured by the FRAP, ABTS, and DPPH assays for the control and LAB-fed queen scallop extracts, respectively. The obtained data illustrate a high correlation between TP and TNF and all antioxidant capacity methods (FRAP, ABTS, and DPPH) for both control and LAB-fed queen scallops. The relationship between the antioxidant capacity and total phenol content was found to be the highest for the control (DPPH: 0.701–ABTS: 0.754) and LAB-fed (DPPH: 0.732–ABTS: 0.819) queen scallop extracts. The correlation coefficient between antioxidant capacity and TNF values varied from 0.489 (DPPH) to 0.628 (FRAP) for control scallops and from 0.077 (FRAP) to 0.727 (ABTS) for LAB-fed scallops. As shown in Table 3, there was a negative correlation between TF and the antioxidant methods ABTS (−0.522) and DPPH (−0.536) in control queen scallops, and in LAB-fed scallops for the FRAP (−0.161) and ABTS (−0.034) assays.

## 4. Discussion

The biopotential of the queen scallop *A. opercularis* has not been investigated to date. Thus, the aim of this study was to assess the phenolic content and AC of the queen scallop, kept in captivity for 30 days, and compare the results with the group of bivalves whose diet was enriched with *L. plantarum* I. The highest obtained value for TP of queen scallop extracts in this study was 67.15 mg GAE/100 g, with water used as the solvent. The obtained TP content was found to be in line with that already published for another marine species, the marine snail *Trochus erithreus*. Zayed et al. [26] showed that ethyl alcohol extract from the marine snail *T. erithreus* had a phenolic content of 213.90 ± 4.88 mg GAE/g extract. However, Mamelona et al. [36] showed that the TP content for the sea cucumber *Cucumaria frondosa* varied from 22.5 to 236.0 mg GAE/100 g DW. Furthermore, several studies have already proven that TP and AC are species-specific, even tissue-specific, and they are different even in some similar species that inhabit different areas in the marine ecosystem [30,37]. Tan et al. [30] discussed that it is possible that organisms living in the intertidal zone have an elevated TP and AC because they are under constant stress and have increased physiological activity, since they are often exposed to air when forced to close their shells. Marine organisms that live deeper are not subjected to such daily stress, and it is possible that this is one of the reasons why they have a slightly lower TP and AC. In addition, subtidal species have less phytoplankton available compared to species that live at the very surface of the sea, and it is well known that the type of organism diet, i.e., phytoplankton rich in various antioxidants, can contribute to a higher TP and AC value [35]. Furthermore, the highest TNF and TF values were obtained in the control group of the queen scallop extracts when 70% ethanol was used as the solvent (46.47 mg GAE/100 g and 31.85 mg GAE/100 g of queen scallop extract tissue, respectively). In addition, the results of this study showed that the TP is positively correlated with TNF, but negatively correlated with TF; thus, it is very likely that TNF is largely responsible for the increased value of TP. Since the TP value was not very high in the investigated queen scallop tissue, especially compared to plant extracts, it is possible that some other molecules also contribute to the observed AC value.

AC assessed by the FRAP assay provided the highest value for the queen scallop extracts when water was used as the solvent (0.19 µM AA/g queen scallop tissue). Mamelona et al. [26] have obtained the antioxidant activity (oxygen radical absorbance capacity, ORAC) for the Atlantic sea cucumber *C. frondose,* whose values were much higher than those found in our study (140 to 800 µmol of Trolox equivalents/g DW). The data for the AC in the shells are scarce and often different protocols are used, making it very difficult for the obtained results to be directly compared. Nevertheless, AC was previously determined for the following bivalves: *Amusium japonicum taiwanicum*, *Atrina pectinata*, *Perna viridis*, *Ruditapes philippinarum*, and *Scapharca kagoshimensis* [30]. In that study, the authors assumed that the AC of bivalves inhabiting the intertidal zone could be higher compared to the bivalves that live on the seabed. This explanation could also be taken for our quite low values of AC for the queen scallop, especially those obtained by the FRAP assay (0.10–0.20 µM AA/g).

The correlation between the FRAP, ABTS, and DPPH assays was found to be significant for TP and TNF (Table 3) for both tested groups of bivalves (control and LAB+ groups). Only the TF value showed a negative correlation with all three tested assays, thus showing that TF might not be a major contributor to the AC of the queen scallop *A. opercularis*. Similar to our results, Goh et al. [35] have found, using the FRAP assay, that phenolic compounds were not a major contributor to the antioxidant capacities of the tested microalgae species (*Chaetoceros* sp. and *Nannochloropsis* sp.). Goh et al. [35] concluded that, although phenolic compounds were present in plant tissue at high concentrations, this might not be true for the microalgae. Furthermore, it can be assumed from the research that this may also be the situation for the bivalves. Some other antioxidants, such as polyunsaturated fatty acids, carotenoids, and polysaccharides that are present in microalgae (the food of the bivalves), can contribute to the AC of the queen scallop [37]. Another explanation for the lower level of total phenols found in this study, compared to the TP content usually found in plant tissue due to different stressors, could be that the bivalves cultured in controlled conditions were fed regularly, thus not being under any stress after the acclimatization period [38]. Contrary to our findings, Mamelona et al. [26] demonstrated that flavonoids are mainly responsible for the antioxidant activity in the sea cucumber *C. frondosa*.

The phenolic content and AC depend on the solvent used for tissue homogenization during the extraction procedure [39]. Goh et al. [35] suggested that different solvents used for the extraction contain different antioxidant compounds that are able to scavenge free radicals. Polar solvents are the most used; for example, water is the highest polarity solvent, 90% methanol is a medium polarity solvent, and 70% ethanol is the lowest polarity solvent [62,64]. There is a need to investigate the effectiveness of different solvents used in the extraction procedures for the determination of the antioxidant capacity. Thus, all three solvents were tested in this study. Water, being a cheap, non-hazardous polar solvent, has been shown to be effective in the extraction of different phenolic compounds from plants [13,40,41]. Furthermore, the powder squid ink was tested for AC with the DPPH and FRAP assays, and both methods showed that water extracts yielded the highest value, followed by 70% ethanol and hexane extracts [42]. Our results confirmed those reached in previous studies, since water extracts of the queen scallops had the highest TP and TNF contents. For the different assays used to obtain the AC value, water as the solvent showed the highest results for all three tested assays, with the ABTS assay showing the highest value in water. This result is also satisfactory for the consumers of shellfish since the bivalves are often prepared for consumption by briefly boiling them in water. It is convenient to know that by preparing shellfish in an aqueous solution, the AC of this species is retained, and the bivalve can be used as food with antioxidants. In addition, the human body is about 60% water, which in this research proved to be the solvent that best retains the AC value. Contrary to our findings, Krishnamoorthy et al. [28] proved that methanolic extracts of the Malaysian green mussel *Perna viridis* showed better AC values if the ABTS and DPPH assays were used, compared to water and ethanol extracts. Furthermore, in their research, ethanolic extracts displayed the highest antioxidant activity in the FRAP assay, whereas in our research, the FRAP assay yielded very similar results for all three solvents tested (water, 70% ethanol, and 90% methanol). Additionally, in our study, even the differences between the control group and the LAB-enriched diet bivalve extracts showed a statistically significant difference only for the 90% methanol extract, in favor of the probiotics-enriched diet. The explanation could be that the AC is not the same in different marine bivalves and the presence of different concentrations of bioactive alkaloids, peptides, phenolic compounds, reducing sugars, and trace elements contribute to AC. Moreover, in one study, the methanolic extract from the marine snail *Hemifusus colosseus* had significantly higher amounts of total phenolic compounds, antioxidant potentiality in free-radical scavenging, and reduced power activity compared to the water extract [42].

The increase in research examining the effect of different probiotic cultures is growing due to the rising resistance of pathogenic organisms to antibiotics. In the future, the use of antibiotics could largely be replaced by the use of probiotic species isolated from, for example, the digestive tract of the same organism in whose diet the probiotic culture will be applied. Our previous research showed that the addition of *L. plantarum* I to the diet of the queen scallop *A. opercularis* (from which LAB was first isolated) enhanced its growth performance and serves as a feasible approach to reduce pathogen levels in aquaculture of this organism [9]. It has previously been shown that *L. plantarum* has very good adhesion properties in the rat intestine, which could also be true for the intestine of other animals [65]. Furthermore, Dell’Anno et al. [6] confirmed the positive effect of the use of two probiotic species, *L. plantarum* and *Lactobacillus reuteri*, as functional feed additives for the prevention of diarrhea in weaned piglets. In this study, when the diet of queen scallops was enriched with the probiotic strain *L. plantarum* I, the phenolic content (TP, TNF, and TF) was often noted to be different if compared with the control group (fed only with phytoplankton), or in some cases a little higher than in the control group (but not statistically significant). The same results were obtained for different AC assays. It is likely that the 30-day duration of the experiment was too short for a statistically significant positive effect of the addition of probiotics in the culture to be achieved. Our assumption is that the AC would show a significant difference between the control group of shellfish and those in which the probiotic was added to the diet if the research was extended to a period longer than 30 days. Furthermore, it should be noted that the FRAP assay showed the highest AC of bivalves whose diet was supplemented with probiotic culture. Namely, in water and 70% ethanol solvents, the FRAP values of control bivalves and those whose diet was supplemented with probiotics were not different, while the 90% methanol extracts of bivalves showed a statistically significant difference precisely in favor of those bivalves whose diet was supplemented with probiotic culture.

## 5. Conclusions

Our results showed, for the first time, that the bivalve *A. opercularis* has antioxidant potential, thus being seafood of great commercial and beneficial value to human health. Furthermore, in this study, the effect of *L. plantarum* I and different types of solvents on the phenolic content and antioxidant capacity of the bivalve queen scallop *A. opercularis* was assessed. Our study revealed that water extracts from queen scallops possessed the highest phenolic content and AC. Furthermore, when bivalves were fed a diet enriched with *Lactiplantibacillus plantarum* I, they demonstrated significantly improved AC across all extract types, as assessed by the FRAP assay. Importantly, a meaningful correlation was identified between AC and TP and TNF levels in both the control group and the *Lactiplantibacillus plantarum* I-treated scallops, underscoring the potential health benefits associated with this dietary enhancement. Further research needs to be carried out, especially in assessing how this bivalve reacts to temperature and pH changes. The hypothesis could be that probiotics could help the shells to better tolerate the climate changes that await us.

## Figures and Tables

**Table 1:** Factorial ANOVA results testing differences in total phenolic content (TP, mg GAE/100 g), total non-flavonoids (TNF, mg GAE/100 g), and total flavonoids (TF, mg CE/100 g) between control scallops and scallops whose diet was enriched with *Lactiplantibacillus plantarum* I. Significant results are marked with an asterisk (*).

Scheme 2		Mean Squares (s^2^)		
Phenolic Content	df	TP	TNF	TF
Diet	1	4.13	6.19	543.98 *
Solvent	2	2996.36 ***	901.97 **	8381.62 ***
LAB*solvent	2	54.80	3.39	1934.03 **

* p < 0.05; ** *p* < 0.001; *** *p* < 0.0001.

**Table 2:** Factorial ANOVA results testing differences in antioxidant capacity measured by the FRAP (µM AA/g), ABTS (µM AA/g), and DPPH (µM AA/g) assays between control scallops and scallops fed with a *Lactiplantibacillus plantarum* 1 (LAB)-enriched diet (Diet), and aqueous solvents. Significant results are marked with an asterisk (*).

Source of Variation		Mean Squares (s^2^)		
Phenolic Content	df	FRAP	ABTS	DPPH
Diet	1	0.006 *	28.81 **	3.48 **
Solvent	2	0.004 *	0.003	0.76 **
LAB*solvent	2	0.001	0.14	1.78 **

* p < 0.05; ** *p* < 0.0001.

**Table 3:** Correlation matrix between antioxidant capacity (FRAP, ABTS, and DPPH assays) and total phenols (TP), total non-flavonoids (TNF), and total flavonoids (TF) contents in control scallops and scallops fed with a *Lactiplantibacillus plantarum* I-enriched diet (LAB). Significant correlations are marked with an asterisk (*).

		Antioxidant Capacity		
Phenolic Content	df	FRAP	ABTS	DPPH
TP	Control	0.591 *	0.754 *	0.701 *
	LAB	0.282	0.819 *	0.732 *
TNF	Control	0.628 *	0.546 *	0.489 *
	LAB	0.077	0.727 *	0.604 *
TF	Control	0.168	−0.522 *	−0.536 *
	LAB	−0.161	−0.034	0.225

* *p* < 0.05.

## Data Availability

The data that support the findings of the present study are available from the corresponding authors upon request.
